# Enhancing the Catalytic Performance of PdNPs for Cr(VI) Reduction by Increasing Pd(0) Content

**DOI:** 10.3390/microorganisms13061346

**Published:** 2025-06-10

**Authors:** Hongfei Lai, Ling Tan, Zhenkun Shi, Shiyi Huang, Wenjia Yu, Guotong Wei, Jianping Xie, Shuang Zhou, Chaoyu Tian

**Affiliations:** 1 Key Laboratory of Ecological Remediation and Safe Utilization of Heavy Metal-Polluted Soils, Hunan Binglang Science Institute, School of Life and Health Sciences, Hunan University of Science and Technology, Xiangtan 411201, China; 2209030512@mail.hnust.edu.cn (H.L.); 24010901003@mail.hnust.edu.cn (S.H.); 2209030415@mail.hnust.edu.cn (W.Y.); 2209030425@mail.hnust.edu.cn (G.W.); zhoushuang@hnust.edu.cn (S.Z.); 2 Biodesign Center, Key Laboratory of Engineering Biology for Low-Carbon Manufacturing, Tianjin Institute of Industrial Biotechnology, Chinese Academy of Sciences, Tianjin 300308, China; zhenkun.shi@tib.cas.cn; 3 School of Minerals Processing and Bioengineering, Central South University, Changsha 410083, China; xiejianping@csu.edu.cn; 4 School of Chemical Engineering, Xiangtan University, Xiangtan 411105, China; tianchaoyu@xtu.edu.cn

**Keywords:** PdNPs, fungal biomass, metallic Pd, Cr(VI) reduction

## Abstract

Hexavalent chromium [Cr(VI)] is a hazardous environmental contaminant, and palladium nanoparticles (PdNPs) have shown promise as catalysts for its reduction. This study explores the primary factor influencing the catalytic performance of PdNPs in Cr(VI) reduction by investigating the crystal structure and composition of PdNPs in fungal-based catalysts. Five Pd-loaded catalysts were synthesized by treating fungal biomass with different chemical reagents, resulting in varying Pd(0) contents. The nanoparticle morphology, chemical states, and functional group interactions during Pd adsorption and reduction were investigated using multiple analytical techniques. The results showed that fungal hyphae remained structurally intact throughout the treatment process. PdNPs smaller than 2 nm were observed, with both Pd(0) and PdO present. The proportion of Pd(0) ranged from 6.4% to 37.2%, depending on the chemical reagent used. In addition, functional groups such as phosphate, amine, hydroxyl, and carboxyl were found to play key roles in palladium binding, underscoring the importance of surface chemistry in the adsorption and reduction process. A strong positive correlation was observed between the Pd(0) content and catalytic activity. Notably, the NCPdSF sample (palladium-loaded biomass treated with sodium formate) exhibited the highest Pd(0) content of 59.2% and achieved the most effective Cr(VI) reduction. These results suggest that Pd(0) content is a key determinant of catalytic efficiency in Cr(VI) reduction and that optimizing chemical treatments to enhance Pd(0) levels can substantially improve catalyst performance.

## 1. Introduction

Chromium (Cr) is a highly toxic heavy metal commonly released into the environment through various industrial processes, such as electroplating, metal processing, leather tanning, textile manufacturing, and the weathering of chrome ores [1,2]. Exposure to high concentrations or prolonged exposure to low levels of chromium poses significant risks to human health, animal health, and the environment safety [3]. Chromium primarily exists in two stable oxidation states: hexavalent chromium (Cr(VI)) and trivalent chromium (Cr(III)). Cr(VI), ranked among the top 20 pollutants on the Superfund Priority List of hazardous elements [4], is markedly more toxic than Cr(III) due to its higher solubility and the formation of highly mobile anionic species (CrO_4_^2−^, Cr_2_O_7_^2−^, and HCrO^4−^) [5]. Consequently, reducing toxic Cr(VI) anions to Cr(III), the less hazardous form, has been recognized as an effective strategy for treating Cr(VI)-contaminated wastewater [6]. Numerous methods have been reported for Cr(VI) removal, including chemical reduction, absorption [7], membrane filtration [8], photocatalytic reduction [9], electrochemical treatment [10], microbiological remediation [11], and ion exchange [12]. However, these approaches are often hindered by limitations such as high operational costs, excessive reagent consumption, and substantial sludge generation [13]. In contrast, catalytic reduction has emerged as a promising alternative due to its simplicity, efficiency, and environmental compatibility, making it an attractive solution for the effective detoxification of Cr(VI) [14].

Metal nanoparticles have garnered significant attention as catalysts for the catalytic reduction of Cr(VI) to Cr(III) due to their high reaction efficiency and selectivity, minimal mass-transfer limitations, and environmentally friendly properties [4]. Nickel nanoparticles synthesized with a three-dimensional yolk-shell-like carbon structure were demonstrated to act as efficient and recyclable catalysts for Cr(VI) reduction, achieving the complete conversion of 100 mg/L toxic Cr(VI) to non-toxic Cr(III) within 30 min [15]. Similarly, cobalt nanoparticles supported on reduced graphene oxide (Co-RGO) exhibited remarkable catalytic activity for Cr(VI) reduction in the presence of formic acid, primarily due to the synergistic interaction between the cobalt nanoparticles and RGO sheets [16]. Under optimized conditions, Co-RGO achieved a Cr(VI) reduction rate of 83.6% within 15 min at an initial Cr(VI) concentration of 160 mg/L [16]. Liu et al. developed silver nanoparticles embedded in biochar (Ag@biochar) by pyrolyzing Ag-contaminated biomass. This material effectively reduced Cr(VI) through a CO-mediated pathway, and complete Cr(VI) reduction was reported within 20 min using formic acid as a reductant at 323 K [17]. Despite their effectiveness, these metal nanoparticles may present limitations such as inadequate stability, diminished redox activity, and potential toxicity concerns [18]. Among various catalytic systems, PdNPs are the primary choice for Cr(VI) reduction due to their unique properties such as well-defined morphology, high intrinsic carrier ability, remarkable stability, and densely populated unsaturated surface coordination sites [19]. For instance, Zhang et al. synthesized uniformly dispersed platinum nanoparticles (size = 5 nm) on polystyrene-b-poly (4-vinylpyridine) nanospheres, which showed high activity and stability (no change after 4 cycles used) as catalysts for Cr(VI) reduction [20]. Pd tetrapods were synthesized through a simple water-based approach involving arginine molecules. They exhibited significantly enhanced catalytic activity for Cr(VI) reduction compared to commercial Pd black because of its larger specific surface area and faster capability in formic acid decomposition [21]. Veerakumar et al. demonstrated the high catalytic performance of PdNPs supported on garlic-derived activated carbon (Pd@GAC) for Cr(VI) reduction. The superior activity of Pd@GAC was attributed to its high specific surface area and absorption capacity, which facilitated effective contact between the substrate and the active catalytic sites [22]. However, the synthesis of PdNPs was influenced by different chemical reagents, which could lead to variations in particle size and morphology. Our previous study revealed that glutaraldehyde could produce PdNPs of different sizes, highlighting the versatility of synthesis methods in tailoring PdNPs for specific catalytic applications [20].

Various methods have been developed to synthesize PdNPs with or without supporting materials. However, the shape, size, surface morphology, chemical composition, particle uniformity and crystal structure are factors that significantly influence their catalytic performance. To identify the critical factor influencing PdNPs’ efficacy in Cr(VI) reduction, five distinct types of catalysts were synthesized and evaluated for their catalytic performance. Specifically, *Neurospora crassa* (*N. crassa*) biomass was employed as a bioabsorbent for Pd(II) ions, followed by chemical treatments using formaldehyde, glutaraldehyde, sodium hydroxide, and sodium formate to produce various PdNPs. The morphological and structural characteristics of the synthesized PdNPs were directly examined using scanning electron microscopy (SEM) and transmission electron microscopy (TEM). The chemical states of palladium in *N. crassa* were further analyzed through X-ray photoelectron spectroscopy (XPS). Additionally, the role of biological components in *N. crassa* related to Pd(II) absorption and PdNP formation was elucidated using Fourier-transform infrared spectroscopy (FTIR). The Cr(VI) removal capabilities of the various catalysts were then investigated to elucidate the influence of PdNPs composition.

## 2. Materials and Methods

### 2.1. Fungus Culture

*Neurospora crassa* (*N. crassa*) was propagated from a potato dextrose agar (PDA) plate into 200 mL liquid PDA medium. The culture was incubated at 30 °C and 200 rpm. After 48 h, the *N. crassa* biomass was harvested by vacuum filtration using filter paper in a Buchner funnel. The collected biomass was washed twice with Milli-Q water to remove residual medium. To obtain a homogeneous biomass suspension, the washed hyphae were mixed with 100 mL of Milli-Q water and homogenized using a milk blender for 30 s. The mixture was then filtered again, and the processed biomass was designated as NC in this study.

For PDA medium preparation, 200 g of potato was boiled in 600 mL of Milli-Q water for 20 min, after which the supernatant was collected by filtration through six layers of gauze. To the supernatant, 20 g of dextrose, 20 g of agar, and a suitable amount of Milli-Q water were added to bring the final volume to 1 L. The liquid PDA medium was prepared similarly by omitting the addition of agar.

### 2.2. Pd-Loaded Sample Preparation

The Pd(II) stock solution was prepared by dissolving sodium tetrachloropalladate(II) (Na_2_PdCl_4_, Sigma-Aldrich, St. Louis, MO, USA) in Milli-Q water. The final pH was adjusted to 3.0 using HCl, and the final concentration was 5 mM.

The Pd(II) absorption was conducted under biomass loading concentration of 20 g/L and at a Pd(II) concentration of 1 mM. Specifically, 100 mL of 5 mM Pd(II) stock solution was mixed with 400 mL of 25 g/L *N. crassa* suspension to obtain the absorption system. The mixtures were then incubated at 30 °C with agitation at 220 rpm. After 3 h absorption, the mixture was evenly divided into 5 portions, each containing 100 mL. The *N. crassa* biomass from each portion were filtered and washed twice with Milli-Q water. The washed and filtered biomass was then used for five distinct treatments:

1. Pd-absorbed *N. crassa* (NCPd): The biomass was freeze-dried.
2. Formaldehyde-treated *N. crassa* (NCPdF): The biomass was suspended in 50 mL of Milli-Q water, and 12 mL of formaldehyde (37–40% concentration) was added to the suspension. The mixture was incubated in 30 °C with agitation at 220 rpm for 12 h. After incubation, the biomass was filtered, washed twice with Milli-Q water, and freeze-dried for subsequent use.
3. Glutaraldehyde-treated *N. crassa* (NCPdG): The biomass was suspended in 50 mL Milli-Q water, and 2.5 mL of glutaraldehyde (50% concentration) was added. The mixture was incubated in 30 °C with agitation at 220 rpm for 12 h. Afterward, the biomass was filtered, washed twice with Milli-Q water, and freeze-dried for subsequent use.
4. Sodium-hydroxide-treated *N. crassa* (NCPdSH): The biomass was suspended in 50 mL Milli-Q water. Then, 2 mL of sodium hydroxide at concentration of 0.3 M was slowly dropped into the suspension under gentle magnetic stirring conditions. The mixture was incubated in 30 °C with agitation at 220 rpm for 12 h. After incubation, the biomass was filtered, washed twice with Milli-Q water, and freeze-dried for subsequent use.
5. Sodium-formate-treated *N. crassa* (NCPdSF): The biomass was suspended in 50 mL degassed sodium formate (25 mM). The mixture was transferred to a serum bottle and flushed with N_2_ for 1 h to drive off the dissolved O_2_ then sealed with butyl rubber stopper. The mixture was incubated in 30 °C with agitation at 220 rpm for 12 h. After incubation, the biomass was filtered, washed twice with Milli-Q water, and freeze-dried for subsequent use.

### 2.3. Quantitative Analysis of Pd in Catalysts

The Pd mass concentrations in the freeze-dried Pd-loaded samples were determined using an inductively coupled plasma (ICP) spectrometer (Shimadzu ICP-7510, Kyoto, Japan) equipped with a polyether-ether-ketone atomizer. Specifically, 0.50 g of each sample (NCPd, NCPdF, NCPdG, NCPdSH, and NCPdSF) was subjected to digestion by adding 10 mL of concentrated nitric acid and 1 mL of perchloric acid. The resulting mixture was heated to 110 °C within 10 min and maintained for 30 min, followed by a temperature increase to 180 °C over 10 min. The lid was removed to allow acid volatilization, and the temperature was maintained at 180 °C until complete dissolution of the sample. Deionized water was then added to bring the final volume to 25 mL for ICP analysis. The experiments were performed in triplicate.

### 2.4. SEM, TEM, XPS, and FTIR Analyses

SEM analysis: The morphologies of freeze-dried samples, including NC, NCPd, NCPdF, NCPdG, NCPdSH, and NCPdSF biomass, were analyzed using SEM. High-resolution images were acquired with a ZEISS Sigma 300 scanning electron microscope (Zeiss, Oberkochen, Germany), operated at an accelerating voltage of 3 kV and a working distance of 4 mm. Prior to imaging, the samples were coated with a thin layer of gold to enhance conductivity.

TEM analysis: TEM analysis was performed on freeze-dried NC, NCPd, NCPdF, NCPdG, NCPdSH, and NCPdSF biomass. The samples were suspended in Milli-Q water, and 20 µL of the suspension was deposited onto Formvar carbon-coated 200-mesh copper grids. The grids were incubated at room temperature for 10 min to facilitate material attachment. Excess suspension was removed using lens wiping tissue, and the grids were air-dried before imaging. Whole-mount cells were visualized using a JEOL JEM-F200 transmission electron microscope (JEOL, Tokyo, Japan) operating at an accelerating voltage of 80 kV. The size of the nanoparticles observed in the HRTEM images was analyzed using ImageJ 1.53 software. Briefly, the scale bar was used to calibrate the relationship between image dimensions and actual size. The diameters of individual PdNPs were measured manually. A total of 35 nanoparticles were analyzed to calculate the average particle size and the corresponding standard error.

XPS analysis: XPS analysis was conducted to study the freeze-dried NCPd, NCPdF, NCPdG, NCPdSH, and NCPdSF biomass. The spectra were obtained using a Kratos Axis Ultra DLD spectrometer (Kratos Analytical, Kyoto, Japan) equipped with a monochromated Al Kα X-ray source (hν = 1486.6 eV). Survey (wide) scans were taken at an analyzer pass energy of 160 eV and multiplex (narrow) high resolution scans at 20 eV. Survey scans were carried out over a 1200–0 eV binding energy range with 1.0 eV steps and a dwell time of 100 ms. Narrow high-resolution scans were run with 0.05 eV steps and 250 ms dwell time. Base pressure in the analysis chamber was 1.0 × 10^−9^ torr and during sample analysis 1.0 × 10^−8^ torr. To ensure consistency across samples, the binding energy scale was calibrated using the C 1s peak at 284.8 eV, corresponding to adventitious carbon. Atomic concentrations were calculated using the CasaXPS version 2.3.14 software and a Shirley baseline with Kratos library Relative Sensitivity Factors (RSFs). Peak fitting of the high-resolution data was also carried out using the CasaXPS software.

FTIR analysis: FTIR spectroscopy was employed to analyze the freeze-dried NC, NCPd, NCPdF, NCPdG, NCPdSH, and NCPdSF biomass. The FTIR spectra were recorded using a Thermo Scientific Nicolet iS20 spectrometer (Madison, WI, USA).

### 2.5. Catalytic Performance of Pd-Loaded Fungus in Cr(VI) Reduction

The freeze-dried NC, NCPd, NCPdF, NCPdG, NCPdSH, and NCPdSF biomass were utilized as catalysts for Cr(VI) reduction. Standardized Cr(VI) solution with a concentration of 20 mM was prepared by dissolving sodium chromate (Na_2_CrO_4_, Sigma-Aldrich) in Milli-Q water. Catalyst suspensions of fungal biomass were prepared by dispersing 10 mg of each material in Milli-Q water to achieve a final concentration of 1 mg/mL. To ensure uniform dispersion, the suspensions were sonicated in an ultrasonic washer for 60 min prior to use.

For Cr(VI) reduction experiments, a reaction mixture was prepared in a cuvette comprising 40 μL of 20 mM Cr(VI) solution, 1660 μL of 30 mM HEPES buffer to maintain a neutral pH, and 100 μL of 1 mg/mL catalyst suspension. The reaction was initiated by adding 200 μL of 10% formic acid. All experiments were conducted in triplicate at 30 °C. For Pd-loaded fungal biomass, the progress of Cr(VI) reduction was monitored by measuring the absorbance at 362 nm using UV-Vis spectrophotometry (2 cm pathlength quartz cuvette). A standard calibration curve was established using Cr(VI) solutions of known concentrations (0.2, 0.4, 0.6, 0.8, and 1.0 mM) to ensure accurate quantification. Each measurement represents the average of three independent replicates. Absorbance was recorded at specific time intervals: 0, 1, 2, 4, 6, 8, 12, 16, 20, 24, 28, 32, 36, 40, 44, 48, 52, 56, and 60 min. After 60 min of reaction, the catalysts were separated from the mixture via centrifugation at 10,000 rpm for 5 min to achieve solid–liquid separation. The resulting catalyst pellets were subsequently reintroduced into a fresh 2 mL reaction system as described above. The catalysts were subjected to five consecutive reuse cycles, each lasting 60 min, and the residual Cr(VI) concentration was determined using UV-Vis spectrophotometry at 362 nm.

The Cr(VI) removal efficiency was calculated using the following equation:

Removal efficiency (%) = (*A*_0_ − *A_t_*)/*A*_0_ (1)

where *A*_0_ is the absorbance at time 0, and *A_t_* is the absorbance at time *t*.

## 3. Results and Discussion

### 3.1. SEM Analysis—Morphological Characteristics

**Figure 1:**
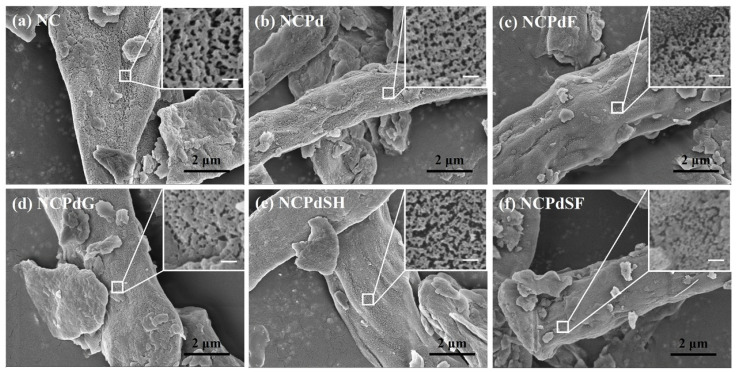
Secondary electron SEM micrographs for different fungal cells (**a**) NC, (**b**) NCPd, (**c**) NCPdF, (**d**) NCPdG, (**e**) NCPdSH, (**f**) NCPdSF. Insets in the upper right corner of each image represent magnified views with a scale bar of 100 nm.

The surface morphologies of NC, NCPd, NCPdF, NCPdG, NCPdSH, and NCPdSF biomass were analyzed using the SEM technique. As shown in Figure 1, cell debris was scattered across all samples, indicating that the hyphae of *N. crassa* were disrupted after homogenization using a milk blender. The hyphae used for Pd absorption were observed with a width of 3–5 μm. The inset images revealed a porous surface structure on the hyphae, likely resulting from the freeze-drying process, which drained the cell envelope and exposed the cellular skeleton. This porous structure might have enhanced Pd absorption and facilitated subsequent chemical treatment processes. No apparent PdNPs were detected on the surfaces of NCPd, NCPdF, NCPdG, NCPdSH, and NCPdSF samples (Figure 1b–f). This absence could be attributed to the detection limitations of the SEM technique since the PdNPs formed on cell surface could be smaller than 2 nm [20]. In comparison to NC biomass, the Pd-loaded sample suggested that neither Pd absorption nor subsequent chemical treatments (e.g., formaldehyde, glutaraldehyde, sodium hydroxide, or sodium formate treatments) caused significant structural disruption to the hyphae.

### 3.2. TEM—PdNPs in Whole Fungal Cells

**Figure 2:**
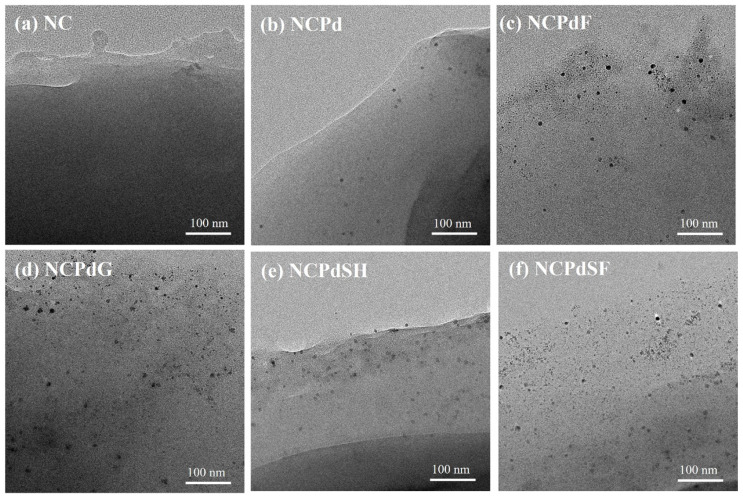
TEM whole-mount micrographs of PdNPs in different fungal cells. (**a**) NC, (**b**) NCPd, (**c**) NCPdF, (**d**) NCPdG, (**e**) NCPdSH, (**f**) NCPdSF.

PdNPs in the freeze-dried NC, NCPd, NCPdF, NCPdG, NCPdSF, and NCPdSH biomass were examined using TEM. As a control, no discernible PdNPs were detected in the NC biomass (Figure 2a). After Pd absorption, a small quantity of PdNPs, ranging from 3 to 8 nm with an average diameter of 5.7 ± 1.4 nm, was observed in the cell envelope of NCPd biomass (Figure 2b). The presence of PdNPs in NCPd suggested that fungal biomass possessed an inherent reduction capability for Pd(II). Previous studies have demonstrated that the outer membrane *c*-type cytochromes and hydrogenase enzymes in the biomass were responsible for the biosynthesis of PdNPs [23]. Functional groups [24], such as -OH, -SH [25], -COOH, and -NH_2_, present on the proteins of the cell wall, played a crucial role in facilitating the reduction of Pd(II) ions [26,27]. The large PdNPs formed after Pd(II) absorption in the NCPdF, NCPdG, NCPdSH, and NCPdSF samples had sizes of 6.2 ± 1.3 nm, 8.04 ± 1.5 nm, 8.14 ± 1.3 nm, and 6.72 ± 1.45 nm, respectively. Furthermore, a significantly higher density of small PdNPs (<2 nm) was observed in these samples (Figure 2c–f), indicating that chemical treatment enhanced PdNP formation. Our previous study demonstrated that Pd-loaded *Cupriavidus metallidurans* CH34 cells produced fine PdNPs smaller than 2 nm after sodium formate treatment, whereas larger PdNPs (10–20 nm) were formed after glutaraldehyde treatment [20]. Similarly, in Pd-loaded yeast, numerous newly synthesized PdNPs (<5 nm) were observed within the periplasm of YPdG cells when glutaraldehyde fixation was applied [28]. These findings collectively highlight that the size of biosynthesized PdNPs was strongly influenced by the microorganism species [29]. Moreover, chemical reagents such as formaldehyde, glutaraldehyde, sodium hydroxide, and sodium formate could facilitate PdNP synthesis by reacting with Pd(II) ions absorbed onto NCPd biomass.

### 3.3. HRTEM—PdNP Crystal Structure in Whole Fungal Cells

**Figure 3:**
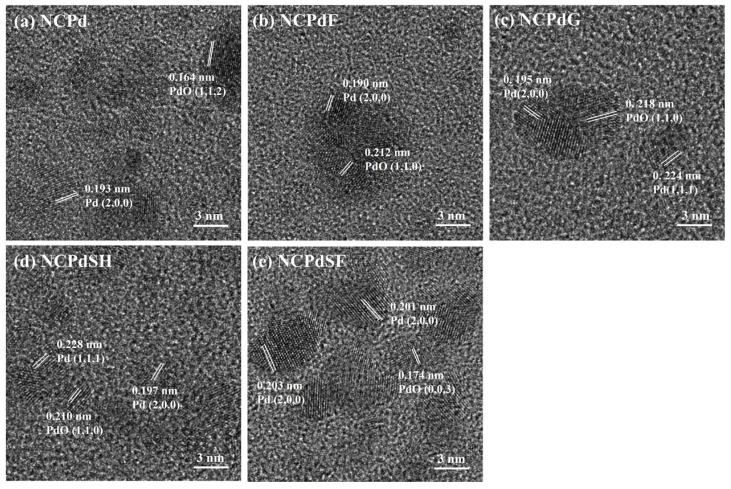
HRTEM images and interplanar spacing of PdNPs in different fungal cells. (**a**) NCPd, (**b**) NCPdF, (**c**) NCPdG, (**d**) NCPdSH, (**e**) NCPdSF.

HRTEM analysis of the Pd-loaded samples, providing insights into the lattice arrangement of PdNPs, is presented in Figure 3. In the NCPd sample, distinct lattice fringes with an interplanar spacing of 0.193 nm were observed (Figure 3a), corresponding to the (2, 0, 0) plane of metallic Pd [30]. Additionally, lattice fringes with an interplanar spacing of 0.164 nm were assigned to the (1, 1, 2) planes of PdO [31], confirming the formation of PdO in *N. crassa* after Pd absorption. In the NCPdF sample (Figure 3b), the nanoparticles exhibited distinct lattice fringes with the interplanar spacing of 0.190 nm, pointing to the (2, 0, 0) plane of metallic Pd (JCPDS No. 87-0638). Furthermore, lattice fringes with an interplanar spacing of 0.212 nm, attributed to the (1, 1, 0) plane of PdO (JCPDS No. 75-0200) [32], were identified. The NCPdG sample (Figure 3c) exhibited an interplanar spacing of 0.218 nm, corresponding to the (1, 0, 0) plane of PdO (JCPDS No. 85-0624). D-spacings of 0.195 nm and 0.224 nm were assigned to the (2, 0, 0) and (1, 1, 1) crystal planes of face-centered cubic Pd, respectively. In the NCPdSH sample (Figure 3d), the interplanar spacing of the 0.197 nm and 0.228 nm were in agreement with the lattice fringes of the (2, 0, 0) and (1, 1, 1) planes of metallic Pd. Furthermore, lattice fringes with an interplanar spacing of 0.210 nm were observed in the substrate, which was consistent with the (1, 1, 0) plane of PdO (JCPDS No. 88-2434). In the NCPdSF sample (Figure 3e), the lattice fringes were observed to have d-spacings of 0.203 and 0.201 nm, which were close enough to the interplanar spacing of the (2, 0, 0) plane in Pd nanoparticles [33] (JCPDS no. 87-0637). The well-resolved fringe pattern of the PdNPs revealed d-spacings of 0.174 nm, corresponding to the (0, 0, 3) interplanar distance of PdO (JCPDS no. 75-0200). Collectively, these observations confirmed the coexistence of metallic Pd and PdO nanoparticles in all Pd-loaded samples, demonstrating the ability of *N. crassa* biomass to reduce Pd(II) to Pd(0).

### 3.4. XPS—Pd Chemical State Analysis

**Figure 4:**
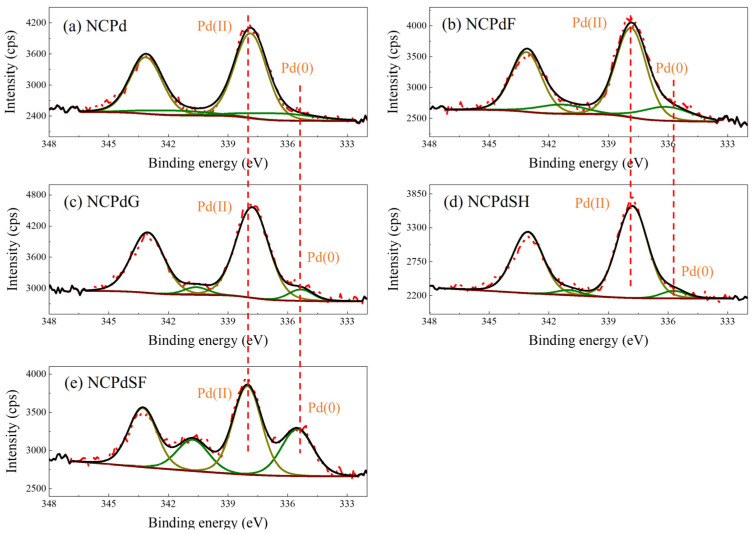
High resolution XPS spectra of Pd 3d from PdNPs in different fungal cells. (**a**) NCPd, (**b**) NCPdF, (**c**) NCPdG, (**d**) NCPdSH, (**e**) NCPdSF. The curves in olive represent Pd(0), while the curves in dark yellow represent Pd(II).

XPS was employed to investigate the electronic states of PdNPs supported on Pd-loaded samples (NCPd, NCPdF, NCPdG, NCPdSF, and NCPdSH). According to previous studies [34,35,36], palladium species exhibited two characteristic peaks corresponding to the Pd_3d5/2_ and Pd_3d3/2_ transition. For bulk metallic palladium (Pd(0)), these peaks were typically observed at binding energies of 336.5 eV (±0.4 eV) and 341.8 eV (±0.4 eV), respectively. The peaks were centered at 337.8 eV (±0.4 eV) and 343.1 eV (±0.4 eV) for Pd(II). In all Pd-loaded biomass samples, the spin-orbit doublet peaks at 337.8 eV and 343.1 eV were attributed to Pd_3d5/2_ and Pd_3d3/2_ of Pd(II). High-resolution Pd 3d spectra of NCPd (Figure 4a) and NCPdF (Figure 4b) revealed double peaks at 336.4 eV and 341.7 eV, corresponding to Pd_3d5/2_ and Pd_3d3/2_ of metallic Pd(0). In NCPdG, NCPdSH, and NCPdSF (Figure 4c–e), slight decreases in the binding energies of Pd(0) were observed, shifting from 336.2 ± 0.2 eV to 335.5 ± 0.2 eV and from 341.5 ± 0.2 eV to 340.7 ± 0.2 eV. These shifts were likely due to changes in the palladium atomic environment [20,28]. In this study, the peak area corresponding to Pd(II) was significantly larger than that of Pd(0), indicating that the majority of palladium on the Pd-loaded biomass existed predominantly in the form of Pd(II). The peak area ratios of Pd(II) to Pd(0) in NCPd, NCPdF, NCPdG, NCPdSH, and NCPdSF were 5.49:1, 4.78:1, 4.63:1, 4.63:1, and 1.69:1, respectively. The ICP analysis indicated that the Pd-loaded samples exhibited similar Pd mass concentrations, which were 2.394 ± 0.034% for NCPd, 2.276 ± 0.016% for NCPdF, 2.372 ± 0.003% for NCPdG, 2.462 ± 0.008% for NCPdSH, and 2.274 ± 0.011% for NCPdSF. However, the Pd(0) content varied depending on the chemical reagent used. For instance, the Pd(0) content was 37.2% in NCPdSF, compared to 15.4% in NCPd. This result suggested that sodium formate facilitated the reduction of absorbed Pd(II) to metallic Pd(0) in NCPd. NCPdSH exhibited the lowest Pd(0) content of 6.4%, indicating an increased Pd(II) content, likely resulting from the formation of Pd(OH)_2_ nanoparticles.

### 3.5. FTIR—Functional Groups Related to Pd(II) Absorption and Reduction

**Figure 5:**
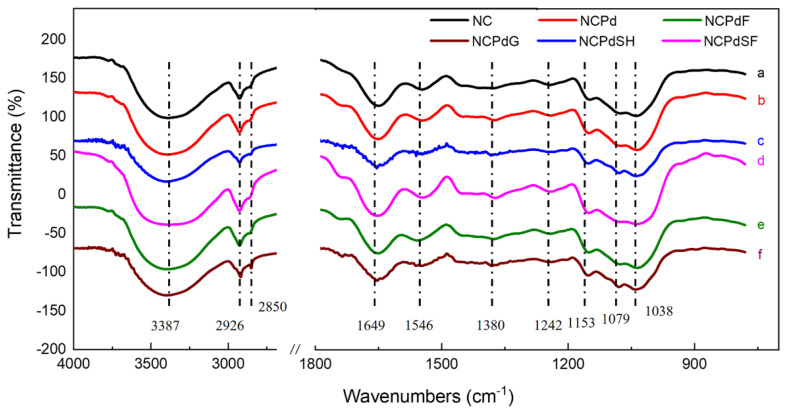
FTIR spectra of (a) NC, (b) NCPd, (c) NCPdF, (d) NCPdG, (e) NCPdSH, (f) NCPdSF. Note, wavenumbers from 800 to 1800 cm^−1^ was amplified.

The infrared spectra FTIR of blank and Pd-loaded *N. crassa* biomass were recorded over the range of 400–4000 cm^−1^ (Figure 5). For blank *N. crassa* biomass, a broad peak at 3387 cm^−1^ was observed, characteristic of hydroxyl (O-H) and N-H stretching vibrations associated with amino groups [37]. The band at 2926 cm^−1^ corresponded to the asymmetric stretching of methylene (C-H) groups in -CH_3_ and -CH_2_ functional groups [38]. A prominent band at 1649 cm^−1^ arose from C-O stretching vibrations conjugated to N-H deformation, indicative of the amide-I band [28,39]. Another band associated with the amide group was present at 1546 cm^−1^, attributed to N-H bond stretching in amide-II [40,41]. The peak at 1380 cm^−1^ corresponded to the symmetric stretching of the C=O bond in COO⁻ groups [42]. The absorption peak at 1242 cm^−1^ was attributed to P=O bonds of polysaccharides [43]. Additionally, the peak at 1038 cm^−1^ could be associated with the stretching vibrations of the C-O-C and C-O groups [44].

For all Pd-loaded samples (NCPd, NCPdF, NCPdG, NCPdSH, and NCPdSF), a characteristic absorption peak of phosphate groups at approximately 1150 cm^−1^ (P=O stretching) was observed, indicating the involvement of phosphate groups in Pd absorption [45]. After Pd absorption (NCPd), the peaks originally observed at 3387 and 1380 cm^−1^ in the blank biomass shifted to 3390 and 1374 cm^−1^, respectively, suggesting the participation of amine, hydroxyl, and carboxyl groups in Pd absorption. It was suggested that the chemical interaction between the Pd ions and the surface functional groups of biomass (*N. crassa*) was the major sorption mechanism [46]. For the formaldehyde- and glutaraldehyde-treated samples, peak shifts associated with hydroxyl (3390 and 3404 cm^−1^), amines (1650 and 1653 cm^−1^), carboxyl groups (1374 and 1382 cm^−1^), and phosphate groups (1238 and 1241 cm^−1^) were observed in NCPdF and NCPdG, respectively. The observed peak shifts in amine and carboxyl groups confirmed the formation of covalent bonds, arising from the reaction between aldehydic groups in formaldehyde (glutaraldehyde) and amino acids in *N. crassa* biomass. [28,47]. For the NCPdSH sample, a significant shift in the carboxyl group peak (1375 cm^−1^) was observed, which was attributed to acid–base interactions. In the NCPdSF sample, the C-H peak shifted from 2926 to 2919 cm^−1^. Additionally, new peaks at 2850 cm^−1^ (asymmetric stretching of C-H bonds) were observed [48,49], confirming the successful incorporation of sodium formate into the carbon structure of NCPdSF catalyst. These interactions likely enhance the catalytic performance of the catalysts in Cr(VI) reduction by modifying the electronic environment and generating additional active sites [50].

### 3.6. Catalytic Activity of Pd-Loaded Biomass in Cr(VI) Reduction

**Figure 6:**
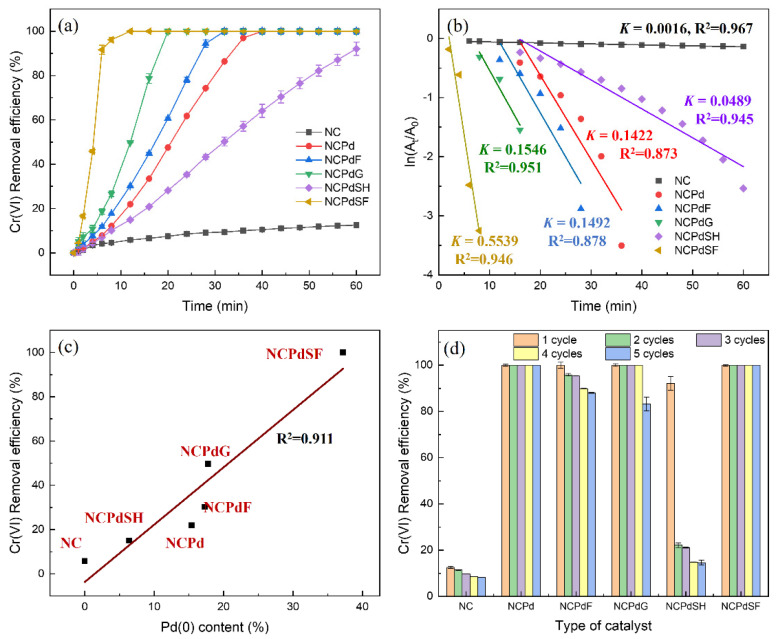
(**a**) Time-dependent Cr(VI) removal efficiency using different catalysts [40 μL of 20 mM Cr(VI) solution, 1660 μL of 30 mM HEPES buffer, 100 μL of 1 mg/mL catalyst suspension, 200 μL of 10% formic acid]; (**b**) the graph of ln(C_t_/C_0_) against reduction time; (**c**) correlation of Cr(VI) removal efficiency with Pd(0) content in different catalysts at 12 min; (**d**) reusability of catalysts in the reduction of Cr(VI) over five consecutive cycles.

The particle size, chemical components, and crystal structure influence the catalytic performance of nanoparticles [20,28]. In this study, various PdNPs were synthesized within *N. crassa* biomass, yielding materials with different Pd(0) contents. To evaluate their catalytic activity, the time-dependent Cr(VI) reduction efficiency was examined for *N. crassa* biomass with different catalysts (Figure 6a). For the blank *N. crassa* sample (NC), the Cr(VI) removal efficiency was approximately 12% at 60 min, attributed to absorption by the biomass. After Pd absorption, the Cr(VI) removal efficiency improved significantly, with complete removal after 40 min for the NCPd sample. Treatments with formaldehyde (NCPdF), glutaraldehyde (NCPdG), and sodium formate (NCPdSF) further enhanced catalytic performance, achieving complete Cr(VI) removal in 32, 20, and 12 min, respectively. In contrast, treatment with sodium hydroxide (NCPdSH) negatively affected the catalytic efficiency of the NCPd sample, resulting in 92% Cr(VI) removal after 60 min. To quantitatively characterize the reaction kinetics and gain deeper insights into the reduction performance, a plot of ln(C_t_/C_0_) versus reduction time for the various catalysts is presented in Figure 6b. The linear correlation observed is indicative of pseudo-first-order kinetics, as demonstrated by the high regression coefficient (R^2^ > 0.873), implying that the catalytic rate of Cr(VI) reduction by PdNPs remains essentially constant over the course of the reaction [51]. Furthermore, rate constants for the NC, NCPd, NCPdF, NCPdG, NCPdSH, and NCPdSF catalysts were determined to be 0.0016, 0.1422, 0.1492, 0.1546, 0.0489, and 0.5539 min^−1^, respectively. The rate constant of the NCPdSF catalyst was approximately 346 times higher than that of NC, emphasizing the significant role of PdNPs in enhancing Cr(VI) reduction. As shown in Figure 6c, the catalytic performance of Pd-loaded samples exhibited a positive correlation with Pd(0) content, with a correlation coefficient of 0.911. The NCPdSF sample, which exhibited the highest catalytic activity for Cr(VI) reduction, had the highest Pd(0) content of 37.2%. Conversely, the NCPdSH sample showed the lowest catalytic activity, consistent with its lowest Pd(0) content of 6.4%. The significant decline in catalytic activity observed for NCPdSH was inconsistent with the increased presence of small PdNPs shown in Figure 2f, suggesting that the PdNPs in this sample were not in the Pd(0) state. It was inferred that sodium hydroxide promoted the formation of Pd(OH)_2_ nanoparticles [Pd^2+^ + 2OH^−^ → Pd(OH)_2_], as this species could be rapidly generated at pH over 5 [52]. Since catalyst reusability is a critical factor for practical applications, the catalysts were recovered and tested for up to five operational cycles (Figure 6d). The NCPd and NCPdSF catalysts exhibited complete Cr(VI) removal after five cycles, demonstrating the robust catalytic activity of PdNPs in these samples. A slight reduction (<12%) in Cr(VI) removal efficiency was observed for the NCPdF and NCPdG catalysts over the five cycles. In contrast, the NCPdSH catalyst exhibited a significant decline in performance from the second cycle onward. In NC sample, the Cr(VI) removal efficiency decreased from 12.5% to 8.2% after five cycles.

Notably, the NCPdSF catalysts demonstrated superior catalytic performance compared to most previously reported materials, exhibiting the highest rate constant of 0.5539 min^−1^. Pitchaimani et al. utilized a Pd@GAC catalyst system for Cr(VI) reduction in the presence of formic acid, achieving a rate constant of 0.421 min^−1^ [22]. Similarly, Kadam and Chen reported rate constants of 0.1564 min^−1^ for GSE-PdNPs [53] and 0.1329 min^−1^ for Bio-CuNPs [54] in Cr(VI) reduction. Furthermore, the PdNPs in the NCPdSF catalyst exhibited a smaller particle size (<8 nm) compared to those in previous studies, thereby providing a higher surface area for catalytic reactions. In this study, Cr(VI) reduction by formic acid was enhanced through the catalytic activity of Pd(0) nanoparticles. In the presence of PdNPs, formic acid undergoes direct dehydrogenation (HCOOH → CO_2_ + H_2_), a process significantly facilitated by the reduction of the absorption energy of formic acid on PdNPs [55]. The absorbed H_2_ then reduces Cr(VI) to Cr(III) on the PdNP surface via the reaction (Cr_2_O_7_^2−^ + 8H^+^ + 3H_2_ → 2Cr^3+^ + 7H_2_O) [20]. In contrast, no Cr(VI) reduction was observed in the NC sample, where Cr(VI) removal was primarily attributed to absorption. In Pd-loaded biomass, the PdNPs formed in *N. crassa* facilitate Cr(VI) reduction to Cr(III) through the aforementioned mechanism. Furthermore, Pd(II) absorbed by the fungal biomass can be converted into PdNPs upon chemical treatment, particularly with sodium formate. Consequently, the Cr(VI) reduction capacity is significantly enhanced in the NCPdSF catalyst.

## 4. Conclusions

**Figure 7:**
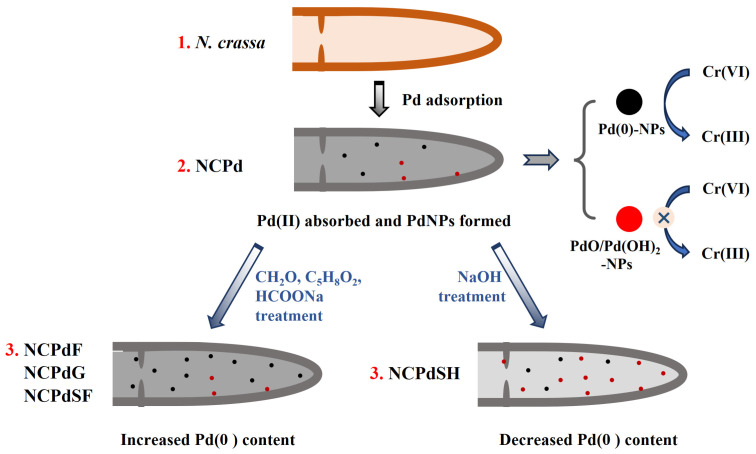
Schematic illustration for PdNP formation in *N. crassa* biomass under different chemical treatment conditions.

The model depicting the formation of various PdNPs on *N. crassa* biomass is presented in Figure 7. In this study, *N. crassa* biomass served as a biosorbent for Pd(II) ions, resulting in the formation of PdNPs. Subsequently, chemical treatments modified the Pd(0) content of Pd-loaded fungal biomass. As a result, the structural integrity of the hyphae was not disrupted either by Pd absorption or chemical treatments. The PdNPS in NCPd were 3–8 nm in size, whereas NCPdF, NCPdG, NCPdSF, and NCPdSH samples exhibited a high density of small PdNPs (<2 nm), indicating enhanced PdNP formation in these samples. Chemical reagents, such as formaldehyde, glutaraldehyde, and sodium formate, enhanced the Pd(0) content in catalysts, while sodium hydroxide treatment reduced the Pd(0) content. FTIR analysis confirmed the involvement of functional groups such as phosphate, amine, hydroxyl, and carboxyl in Pd absorption and the subsequent chemical treatments.

The catalytic reduction of Cr(VI) was significantly influenced by the component of PdNPs. Specifically, metallic Pd(0) played a critical role in facilitating Cr(VI) reduction by using formic acid as a reductant, while PdO/Pd(OH)_2_ nanoparticles lacked this catalytic capability. Consequently, the Cr(VI) reduction performance varied among the catalysts depending on their Pd(0) content. Among the tested catalysts, NCPdSF exhibited the highest removal efficiency, achieving complete removal of Cr(VI) within 12 min. In summary, this study confirmed that the catalytic efficiency of PdNPs in Cr(VI) reduction was predominantly determined by the Pd(0) content in catalysts, with a higher Pd(0) content correlating to superior catalytic performance.

## Data Availability

The original contributions presented in this study are included in the article. Further inquiries can be directed to the corresponding author.
